# Inflammatory cause of metabolic syndrome via brain stress and NF-κB

**DOI:** 10.18632/aging.100431

**Published:** 2012-02-11

**Authors:** Dongsheng Cai, Tiewen Liu

**Affiliations:** Department of Molecular Pharmacology and Diabetes Research Center, Albert Einstein College of Medicine, Bronx, NY 10461

**Keywords:** Brain, hypothalamus, stress, inflammation, NF-κB, metabolic syndrome, disease

## Abstract

Metabolic syndrome, a network of medical disorders that greatly increase the risk for developing metabolic and cardiovascular diseases, has reached epidemic levels in many areas of today's world. Despite this alarming medicare situation, scientific understandings on the root mechanisms of metabolic syndrome are still limited, and such insufficient knowledge contributes to the relative lack of effective treatments or preventions for related diseases. Recent interdisciplinary studies from neuroendocrinology and neuroimmunology fields have revealed that overnutrition can trigger intracellular stresses to cause inflammatory changes mediated by molecules that control innate immunity. This type of nutrition-related molecular inflammation in the central nervous system, particularly in the hypothalamus, can form a common pathogenic basis for the induction of various metabolic syndrome components such as obesity, insulin resistance, and hypertension. Proinflammatory NF-κB pathway has been revealed as a key molecular system for pathologic induction of brain inflammation, which translates overnutrition and resulting intracellular stresses into central neuroendocrine and neural dysregulations of energy, glucose, and cardiovascular homeostasis, collectively leading to metabolic syndrome. This article reviews recent research advances in the neural mechanisms of metabolic syndrome and related diseases from the perspective of pathogenic induction by intracellular stresses and NF-κB pathway of the brain.

## INTRODUCTION

### Brain inflammation in metabolic syndrome

Looking back upon the time when the human species had to spent tremendous efforts to gather enough food for survival, there is no doubt that modern industrialization has succeeded at making many choices of calorie-abundant food easily available with little physical efforts. However, this over-correction of food crisis has led to an opposite pattern of medical problems by introducing a worldwide outbreak of overnutrition-related diseases such as obesity, type 2 diabetes (T2D), and cardiovascular diseases (CVDs) [1-7]. While this overnutrition-featured social economic environment will continue to exist, the health consequences are hefty and can threaten the fundamental welfare of modern humankind [8-12]. From the physiological perspective, outbursts of these health problems are often preceded by a cluster of interconnected pathophysiological abnormalities including obesity, insulin resistance, impaired glucose tolerance, dyslipidemia and high blood pressure, which are collectively called metabolic syndrome [13-19]. Thus, early therapeutic and preventive interventions against metabolic syndrome may represent an economic and effective strategy to control the deleterious outcomes of T2D and CVDs. However, there seems to be little progress in this aspect, largely due to insufficient understandings of the underlying root mechanisms of these disorders. However, cross-field studies from endocrinology and immunology have begun to change this landscape considerably since the last decade. A milestone discovery is that instead of merely being a contributor to energy excess, overnutrition has been recognized as an independent environmental factor that is targeted by innate immune system to trigger an atypical form of inflammation, which leads to metabolic dysfunctions at cellular, organ, and systemic levels [20-33]. Mechanistic studies further showed that such metabolic inflammation is related to the induction of various intracellular stresses such as mitochondrial oxidative stress, endoplasmic reticulum (ER) stress, and autophagy defect under prolonged nutritional excess. More recently, this intracellular stress-inflammation process for metabolic syndrome has been established in the central nervous system (CNS) and particularly in the hypothalamus [28-46]. Importantly, the CNS and the comprised hypothalamus are known to govern various metabolic activities of the body including appetite control, energy expenditure, carbohydrate and lipid metabolism, and blood pressure homeostasis [47-63]. In the following sections, we describe recent research advances that address the roles of brain stress and inflammation in metabolic syndrome and related diseases from molecular, cellular, and physiological perspectives, with a particular emphasis on the comprised hypothalamus.

### Brain oxidative stress and metabolic syndrome

Reactive oxygen species (ROS) refer to a class of radical or non-radical oxygen-containing molecules that have high oxidative reactivity with lipids, proteins, and nucleic acids. There are many potential sources of ROS in cells [64]. Mitochondria are the cellular organelles that generate energy in the form of ATP. However, this process is coupled with production and accumulation of oxidant by-products such as superoxide anions (O2-) within mitochondria and cytoplasm. Thus in quiescent cells, a large measure of intracellular ROS comes from the leakage of mitochondrial electron transport chain (ETC). Another major source of intracellular ROS is the intentional generation of superoxides by nicotinamide adenine dinucleotide phosphate (NADPH) oxidase, which are used by cells for phagocytic defense or normal signaling. In addition, there are other ROS-producing enzymes such as cyclooxygenases, lipoxygenases, xanthine oxidase, and cytochrome p450 enzymes, which are involved with specific metabolic processes. To counteract the toxic effects of molecular oxidation by ROS, cells are equipped with a battery of antioxidant enzymes such as superoxide dismutases, catalase, peroxiredoxins, sulfiredoxin, and aldehyde dehydrogenases. When the production and clearance of ROS is balanced at a physiological homeostatic level, ROS does not pose a threat to cells. In fact, physiological levels of intracellular ROS can be normally involved in certain cell functions, such as membrane ion transport, generation of intracellular Ca2+ wave, activation of protein kinase, and regulation of gene expression [65]. However, when ROS homeostasis is disrupted due to various environmental or pathological factors, excessive ROS are accumulated in the mitochondria and cytoplasm – a condition referred to as intracellular oxidative stress, which can cause oxidative damages of cells and disease consequences [66-71]. Indeed, intracellular oxidative stress has been indicated to contribute to metabolic syndrome and related diseases, including T2D [72; 73], CVDs [74-76], neurodegenerative diseases [69; 77-80], and cancers [3; 81].

The brain utilizes a large amount of oxygen and ATP to support its normal functions, resulting in a high susceptibility to oxidative stress [68;70;71;82]. Indeed, intracellular oxidative stress is highly associated with the development of neurodegenerative diseases [69] and brain aging [83], suggesting that CNS is an important site targeted by oxidative stress. This understanding brings up a question of whether brain oxidative stress could play an important role in the pathogenesis of metabolic diseases, given that the brain and particularly the hypothalamus are the central regulators of whole-body energy and metabolic homeostasis. Despite that research exploration in this regard has been very limited, there is evidence in the literature supporting this hypothesis. For example, dietary obesity was found to induce NADPH oxidase-associated oxidative stress in rat brain [84], indicating that brain oxidative stress could potentially mediate the pathogenesis of overnutrition-related metabolic diseases. Other more direct evidences include that mitochondrial dysfunction in hypothalamic proopiomelanocortin (POMC) neurons causes central glucose sensing impairment [85], and brain mitochondrial dysfunction induced by genetic deletion of peroxisome proliferator-activated receptor coactivator 1α (PGC-1α) disrupts central regulation of energy homeostasis [86]. Thus, the role of brain oxidative stress in the development of metabolic diseases represents a new and highly interesting research topic. Overall, intracellular oxidative stress in the brain is potentially widely implicated in the pathogenesis of metabolic syndrome and related diseases, and defining the molecular and cellular pathways upstream and downstream of brain oxidative stress will significantly advance the mechanistic understandings of these diseases.

### Brain ER stress and metabolic syndrome

Endoplasmic reticulum (ER) is the cellular organelle responsible for protein synthesis, maturation, and trafficking to secretory pathways. Since cellular metabolic demands undergo fluctuations depending on systemic physiological conditions, ER uses its unfolded protein response (UPR) machinery to fine tune its protein synthesis, folding, and secretion accordingly [87]. Three ER membrane-associated protein sensors, PKR-like endoplasmic reticulum kinase (PERK), inositol requiring enzyme-1 (IRE1), and activating transcription factor-6 (ATF6) act as protein sensors to initiate three branches of UPR pathways. When there is no high demand for protein production, these sensors are bound by chaperone protein BiP/GRP78 and stay in an inactive state. However, when there is an increase in the amount of newly synthesized protein or an accumulation of misfolded proteins in ER lumen, BiP is removed, resulting in activation of PERK and IRE1 and their downstream signaling cascades [87]. Activation of ATF6 additionally requires molecular reduction and translocation to Golgi [88;89]. Activated IRE1 produces an active form of transcription factor X-box binding protein-1 (XBP1), which together with ATF6 initiates transcription of genes that promote ER biogenesis, enhance ER folding capacity, promote secretion of ER-associated proteins, and facilitate degradation of misfolded proteins [90;91]. The endoribonuclease activity of IRE1 also directly decreases protein translation by degrading mRNAs [92]. Activation of PERK leads to phosphorylation and activation of eukaryotic translational initiation factor 2α (eIF2α), which reduces global protein synthesis via competitive inhibition of eIF2B transcriptional complex [93]. Through a combination of these mechanisms, UPR can efficiently resolve ER stress and maintain ER homeostasis under physiological conditions.

However, when cellular metabolic challenges that trigger ER stress is severe or persistent as in pathological settings, UPR may not be sufficient to neutralize ER stress, which leads to ER stress-related pathological changes at molecular, cellular, and systemic levels. Such unresolved ER stress can induce cell apoptosis [87;94], which forms the pathogenic basis for neurodegeneration, diabetic islet cell death, atherosclerosis, myocardial infarction, and stroke [95-99]. Alternatively, ER stress can activate cellular inflammatory pathways which impairs cellular functions and leads to metabolic disorders [100]. In addition, ER stress causes cellular accumulation of ROS to induce oxidative stress [101], and oxidative stress reciprocally promotes ER stress by inhibiting ATF6 activation [88], both of which synergistically contribute to the development of metabolic disorders. Indeed, ER stress has been associated to obesity, insulin resistance, T2D, CVDs, cancers, and neurodegenerative diseases [23;95;100;102;103]. Due to the central role of brain in metabolic control, the role of brain ER stress in metabolic disease has come into focus in recent years. Expanding the previous knowledge that brain ER stress underlies neurodegenerative diseases [95], recent studies have causally linked brain ER stress to the development of metabolic syndrome and related disorders such as overeating, obesity, leptin resistance, insulin resistance, β cell dysfunction, and hypertension [34;39; 42;104;105] under conditions of overnutrition [34;42] and related inflammatory insults [105]. These findings have excitingly suggested brain ER stress as a novel therapeutic target for metabolic syndrome, and the underlying molecular basis of brain ER stress will be further discussed in the following relevant sections.

### Brain autophagy defect and metabolic syndrome

Autophagy is an evolutionarily conserved lysosomal degradation pathway that plays essential roles in maintaining cellular homeostasis and promoting cell survival, growth, and differentiation against adverse conditions [106;107]. To maintain a healthy and functional intracellular environment, cells must constantly clean up defective proteins (e.g., misfolded proteins overflowing from ER stress) or damaged organelles (e.g., dysfunctional mitochondria from prolonged oxidative stress). This housekeeping function is carried out by three protein degradative machineries – ubiquitin-proteasome system (UPS), chaperone-mediated autophagy (CMA), and macroautophagy (namely autophagy). UPS generally targets specific short-lived proteins, and CMA only degrades proteins containing particular peptide motifs. By comparison, only autophagy has the capacity for bulk degradation of long-lived cytosolic proteins and whole organelles. In the latter case, special terms such as mitophagy [108], reticulophagy [109], and pexophagy [110] were used to describe autophagy of dysfunctional mitochondria, ER, or excessive peroxisomes, respectively. Under normal growth conditions, autophagy occurs at a basal level to support cell growth, development and differentiation [111-117]. However, under environmental stress such as nutrient deprivation or hypoxia, autophagy is strongly induced to breakdown macromolecules into reusable amino acids and fatty acids for survival. From this perspective, complete loss of autophagy is lethal in experimental mice [114].

Tissue-specific impairment of autophagy, though not lethal, leads to the development of diverse diseases such as infection [118-120], cancer [121;122], muscle disorders [123], heart diseases [124], neurodegenerative diseases [115-117], and aging [125]. A common pathological feature of these diseases is the formation of intracellular aggregates from dysfunctional proteins or organelles [107]. The role of brain autophagy defect in metabolic syndrome has been nearly uninvestigated in the past. However, such prediction is well reasoned. First, studies of autophagy in specific peripheral tissues such as liver [126-129], skeletal muscle [130], and pancreatic β cells [131-133] have implicated autophagy defect in the pathogenesis of metabolic syndrome such as T2D and lipid disorders, with the only exception that autophagy defect in fat cells can impair adipogenesis to counteract fat expansion and the development of obesity [134;135]. Second, autophagy defect in the CNS has been causally linked to a number of neurodegenerative diseases including Alzheimer disease, Parkinson's disease, Huntington's disease, and transmissible spongiform encephalopathies [136-138], indicating an indispensible role of autophagy in maintaining CNS function. Indeed, we recently showed that intact autophagy function is required for the hypothalamus to properly control metabolic and energy homeostasis, while hypothalamic autophagy defect leads to the development of metabolic syndrome such as obesity and insulin resistance [40].

**Figure 1:**
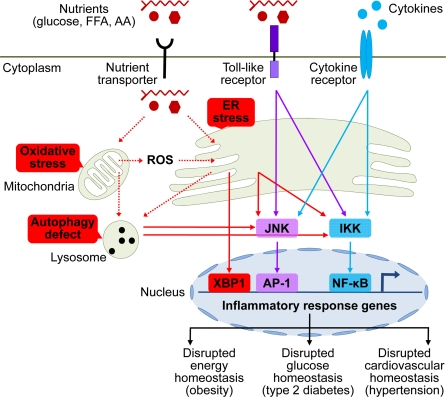
Brain stress and inflammation in the development of metabolic syndrome Overnutrition in the forms of high circulating levels of glucose, free fatty acid (FFA), and amino acids (AA) is the predominant pathogenic inducer of central metabolic inflammation. Excessive nutrients transported into cells can pose severe stresses on cellular metabolic machinery, affecting organelles such as mitochondria and endoplasmic reticulum (ER) which are responsible for nutrient oxidation and protein synthesis, respectively. As a result, intracellular reactive oxygen species (ROS) increase due to heightened mitochondrial activities, leading to intracellular oxidative stress. In parallel, high levels of cellular metabolic activities demand increased protein synthesis and folding by ER, leading to ER stress. Additionally, high levels of intracellular ROS from oxidative stress can escalate ER stress. Prolonged oxidative stress and ER stress can cause intracellular accumulation of dysfunctional mitochondria, ER, and other cytosolic proteins, leading to increased autophagy stress and autophagic defect. All these intracellular stresses are activators of cellular proinflammatory kinases, among which IκB kinase (IKK) and c-Jun N-terminal kinase (JNK) have been implicated. Activation of these proinflammatory pathways leads to transcription of inflammatory response genes via nuclear transcription factors NF-κB and AP-1. ER stress can also directly induce transcription of inflammatory genes via activating transcription factor X-box binding protein-1 (XBP1). Certain extracellular nutrient species can bind to toll-like receptors to activate intracellular proinflammatory signaling. Furthermore, local or systemic inflammatory cytokines can reinforce metabolic inflammation via cytokine receptor signaling. Such collective onset of cellular inflammation impairs normal cellular functions, leading to central dysregulation of various physiological processes across energy balance, glucose tolerance, and cardiovascular homeostasis, which underlies the development of metabolic syndrome and related diseases.

One lingering question is: what are the inducers of hypothalamic autophagy defect in metabolic syndrome? A plausible answer is brain autophagy defect may occur secondarily to oxidative stress and ER stress in the development of central metabolic dysregulations, presumably when intracellular accumulation of damaged mitochondria, ER, and misfolded proteins exceeds the degradative capacity of autophagy machinery. This hypothesis can be inferred from the observation that metabolic disorders related to central autophagy defect are late onset [40], and indeed prolonged oxidative stress or ER stress has been shown to impair autophagy function in disease milieu of cancer or aging [139;140]. But obviously further experimental investigations are needed to draw conclusions.

### Brain immune receptors and metabolic syndrome

In addition to the primary roles of receptor-independent intracellular stress pathways in overnutrition-induced central metabolic dysregulations, immune receptor-mediated pathways can also function in the hypothalamic inflammatory mechanisms of metabolic syndrome. In this regard, toll-like receptor (TLR) pathway has received substantial research attentions. TLRs are an important class of membrane-bound pattern recognition receptors in classical innate immune defense, primarily functioning to promote synthesis and secretion of immune response molecules upon binding by “non-self” molecules (e.g., pathogens) [141;142]. Most hypothalamic cell types including neurons and glia cells express TLRs and thus can mediate innate immune response to local or systemic inflammatory stimuli at least through TLRs [143-145]. In the context of metabolic dysregulations, overnutrition constitutes an environmental stimulus that can activate TLR pathways to mediate the development of metabolic syndrome related disorders such as obesity, insulin resistance, T2D, and atherosclerotic CVDs in rodents [146-157]. Isoforms TLR1, 2, 4, and 6 may be particularly pertinent to pathogenic signaling induced by lipid overnutrition, since these receptors are hyper-responsive to extracellular lipids as shown in studies on adipocytes, macrophages and myocytes [147-150]. The pathogenic significance of TLR signaling in metabolic syndrome has recently been appreciated in the CNS [36;37]. As shown in the literature, hypothalamic TLR4 and downstream inflammatory signaling are activated in response to central lipid excess via direct intra-brain lipid administration or HFD-feeding [36], while overnutrition-induced metabolic derangements such as central leptin resistance, systemic insulin resistance, and weight gain can be significantly prevented in mice with brain-specific inhibition of TLR4 signaling [37]. Furthermore, brain-specific inhibition of TLR4 signaling [37] reproduced the protective effects of whole-body TLR4 deficiency [36;154] against overnutrition. All these evidences based on brain TLR signaling further support the notion that CNS is the primary site for overnutrition to cause the development of metabolic syndrome.

In addition to TLRs, cytokine receptors can also participate in the central induction of metabolic syndrome and related diseases, given that a prominent pathologic feature of these diseases, especially in the late stage, is the prevalent presence of cytokines in the circulation and various tissues of the body [20-30]. These circulating cytokines can limitedly travel to the hypothalamus through the leaky blood-brain barrier around the mediobasal hypothalamus to activate hypothalamic cytokine receptors. In addition to systemic cytokines, local inflammation in the brain induced by intracellular stresses can lead to local production and release of cytokines, which can work on cytokine receptors in adjacent neural cells. Through these combined actions, brain cytokine receptor signaling can help sustain and/or augment brain inflammation to underlie the escalation of metabolic disorders.

Evidences supporting the involvement of cytokine receptors in central metabolic dysregulations mainly come from studies using central administration of cytokines or genetic deletions of cytokines or receptors. However, both approaches have pros and cons. The pharmacological studies, while having the advantage of directly targeting the brain, have major issues with dose usage. Most of the time, the doses applied did not reflect the pathophysiological levels in metabolic syndrome and related diseases, which unintentionally skewed the interpretations of their biological effects. The genetic knockout approach, while providing clean loss-of-function models to infer gene functions, has major limitations with site-specificity, i.e., most cytokine or cytokine receptor knockout models available now target the whole body instead of a specific tissue or cell type. Nonetheless, significant evidences have been recently documented demonstrating the role of cytokine receptor pathways in the development of metabolic syndrome components. For example, central administration of TNF-α at low doses faithfully replicated the effects of central metabolic inflammation in enhancing eating, decreasing energy expenditure [158;159], and causing obesity-related hypertension [38]. Supportively, genetic deficiency of either TNF-α [160;161] or TNF-α receptor [158;162] prevented overnutrition from inducing obesity or insulin resistance in mice. Resistin, an adipocyte-derived proinflammatory cytokine, has been found to promote hepatic insulin resistance through its central actions [163]. On the contrary, anti-inflammatory cytokines such as interleukin-10 or interleukin-6 were found to mediate the metabolic benefits of exercise through reducing hypothalamic inflammation [164]. Taken together, using specific forms, both TLR pathways and cytokine receptor pathways are involved in central inflammatory mechanism of metabolic syndrome and related diseases. Meanwhile, future studies are still needed to delineate the molecular signaling and potential neural cell type-specificity of these programs.

### Brain oxidative stress and IKKβ/NF-κB signaling in metabolic syndrome

Mammalian IκB kinase β (IKKβ) and nuclear factor-κB (NF-κB) comprise a master proinflammatory pathway that has pivotal roles in classical innate immune response [165]. In quiescent state, NF-κB resides in the cytoplasm in an inactive form due to inhibitory binding by IκBα protein. A wide range of extracellular immune stimuli can induce IKKβ activation via receptor-mediated pathway, leading to IκBα phosphorylation and degradation and subsequent release of NF-κB activity. Activated NF-κB enters the nucleus to induce transcription of a myriad of genes that mediate diverse cellular processes such as immunity, inflammation, proliferation, apoptosis, and cellular senescence [166]. Research in the past decade has found that activation of IKKβ/NF-κB proinflammatory pathway in metabolic tissues is a prominent feature of various metabolic disorders related to overnutrition [20-33]. However, this type of inflammation has unique features compared to classical (e.g., pathogen-induced) inflammation. For instance, it happens in metabolic tissues, it is mainly associated with overnutrition-induced metabolic derangements, and most importantly, it is relatively low-grade and chronic. Hence, a special name of “metabolic inflammation” or “metaflammation” is given to refer to this type of atypical inflammation [28;29]. The biological effects of NF-κB-mediated metabolic inflammation are deleterious at cellular and tissue levels, including impairments of normal intracellular signaling and disruptions of metabolic physiology. More recently, this paradigm of IKKβ/NF-κB-mediated metabolic inflammation has been identified in the CNS – particularly the comprised hypothalamus, which primarily accounts for to the development of overnutrition-induced metabolic syndrome and related disorders such as obesity, insulin resistance, T2D, and obesity-related hypertension [28-46]. To understand the pathogenic signaling cascade mediated by IKKβ/NF-κB in the CNS, a keen research effort has been made to elucidate the intracellular changes that bridge overnutrition and hypothalamic NF-κB activation. Albeit not fully developed, existing evidences have pointed to intracellular oxidative stress and mitochondrial dysfunction as upstream events that mediate hypothalamic NF-κB activation in a receptor-independent manner under overnutrition.

NF-κB is a redox-sensitive transcription factor whose activity is affected by cellular oxidative state [64;167]. A major mechanism that oxidative stress can activate NF-κB is through ROS-induced alternative phosphorylation of IκBα which abolishes its inhibition of NF-κB [168-172]. Additionally, oxidative stress can activate NF-κB pathway through oxidative inactivation of NF-κB pathway-related phosphatases such as IKK phosphatases and PTEN (phosphatase and tensin homolog, an Akt phosphatase), which causes upactivation of NF-κB pathway kinases such as IKKs (α, β, and γ) and Akt, leading to NF-κB activation [173-175]. Activated NF-κB can induce cytotoxic products that exacerbate inflammation and oxidative stress and promote apoptosis [176], leading to oxidative stress-induced cell dysfunction or cell death, respectively [64]. In the context of metabolic syndrome, oxidative stress-related NF-κB activation in metabolic tissues or vascular systems has been implicated in a broad range of metabolic syndrome-related diseases, such as diabetes, atherosclerosis, cardiac infarct, stroke, cancer, and aging [177-184]. In the CNS, the potential link between oxidative stress and NF-κB-mediated inflammation in central metabolic dysregulations has not been adequately studied. However, such a mechanistic connection can be reasonably hypothesized based on several lines of evidence. First, overnutrition is an environmental inducer for intracellular oxidative stress regardless of tissues involved [7], because excessive nutrients, when transported into cells, directly increase mitochondrial oxidative workload, which causes increased production of ROS by mitochondrial ETC. Second, oxidative stress has been shown to activate NF-κB pathway in neurons or glial cells in several types of metabolic syndrome-related neural diseases, such as stroke [185], neurodegenerative diseases [186-188], and brain aging [189]. Third, central nutrient excess (e.g., glucose or lipids) has been shown to activate NF-κB in the hypothalamus [34-37] to account for overnutrition-induced central metabolic dysregulations. Additionally, mammalian histone deacetylase Sirtuin 1 has been shown to protect against metabolic syndrome related diseases such as atherosclerosis and aging, and such metabolic protective effects are associated with inhibition of ROS production and suppression of NF-κB activation [190;191]. Thus, intracellular oxidative stress seems to be a likely pathogenic link that bridges overnutrition with NF-κB activation leading to central metabolic dysregulation. Finally, because activation of NF-κB pathway can reciprocally reinforce intracellular oxidative stress through induction of ROS-producing enzymes [176], the vicious cycle between oxidative stress and NF-κB activation may account for the refractory nature of overnutrition-induced metabolic disorders. Future studies of testing the pathogenic model of the connection between oxidative stress and NF-κB in the brain will help form a more complete understanding of the central mechanisms of overnutrition-induced metabolic diseases.

### Brain ER stress and IKKβ/NF-κB signaling in metabolic syndrome

UPR signaling of ER can actively respond to changes of cellular nutrient state such as increased protein synthesis, glucose or energy deprivation, hypoxia, or even elevated intracellular lipid storage [87]. The underlying molecular events of UPR signaling include controlled transcription of many genes that are important for glucose and lipid metabolism [100]. In this context, ER is positioned to coordinate nutrient sensing with metabolism at the cellular level to safeguard systemic metabolic homeostasis. In support of this model, mice that are deficient in UPR pathways, such as PERK-deficient mice [192], eIF2α mutant mice [98;193] or mice with XBP1 haploinsufficiency deficiency [194], are prone to ER stress and develop metabolic disorders such as impaired glucose tolerance, insulin resistance, islet cell dysfunction, and increased adiposity. Along the same vein, overnutrition can present the cell with a metabolic overload that exceeds the physiological adaptive range of UPR, resulting in the development of ER stress and systemic metabolic disorders. Indeed, chronic ER stress in peripheral metabolic tissues such as adipocytes, liver, muscle, and pancreatic cells is a salient feature of overnutrition-related diseases [100;194;195].

Further interests were diverted to how ER stress transduces overnutrition signals into metabolic derangements, and metabolic inflammation was brought upon as a mediator, as all three branches of UPR pathways are linked to proinflammatory IKKβ/NF-κB or JNK signaling [196-199]. However, it is difficult to mechanistically dissect the relationship between overnutrition, ER stress, and inflammation in peripheral tissues using animals that have already developed these abnormalities under chronic overnutrition, because obesity (the most common outcome of chronic overnutrition) and the associated systemic inflammation by themselves are potent inducers of ER stress. By contrast, this question has been well addressed in the CNS. Two recent studies showed that overnutrition induces ER stress as well as IKKβ/NF-κB activation in the hypothalamus of mice fed with a high-fat diet (HFD) [34], and central administration of ER stress inducer mimicked HFD feeding to activate hypothalamic NF-κB in mice on a normal chow diet [34;39]. More importantly, intra-third ventricle infusion of ER stress inhibitor suppressed the activation of hypothalamic NF-κB by HFD feeding [34], demonstrating that ER stress can act as a downstream effector of overnutrition to induce NF-κB-mediated inflammation in the brain. In the meanwhile, a sustained development of ER stress appears to depend on IKKβ/NF-κB pathway activity, because neither HFD feeding nor central administration of chemical ER stress inducer was able to induce hypothalamic ER stress in mice with central inhibition of IKKβ/NF-κB pathway [34;39]. Further bolstering the reinforcing effect of NF-κB activation on ER stress, TNF-α, a product of NF-κB activation, was shown to induce ER stress in the hypothalamus, although the extent of stress induction by TNF-α itself was less than complete [105]. Finally, ER stress can indirectly promote inflammation via induction of oxidative stress [100;101]. Altogether, recent literature supports a model that brain ER stress and NF-κB activation reciprocally promote each other in the development of central metabolic dysregulations. Future studies may reveal more proinflammatory kinases associated with brain ER stress signaling under overnutrition, and different brain cell types may preferentially employ different signaling cascades in disease development.

### Brain autophagy defect and IKKβ/NF-κB signaling in metabolic syndrome

As a stress-response mechanism against adverse growth conditions such as nutrient depletion, and as a machinery to maintain normal intracellular environment, autophagy is key to cell/organism survival and proper functioning [106;107]. Unsurprisingly, autophagy defect has been linked to the development of a number of systemic diseases [115-125] including metabolic syndrome, T2D, and lipid abnormalities [126-133;200]. Of note, in the majority of these cases, the underlying pathogenesis lies in the failure of autophagy machinery to efficiently remove defective proteins or damaged organelles from the cytosol [107]. In terms of the pathogenic root of autophagy defect, chronic intracellular stress and particularly ER stress seem to be the critical upstream event. Indeed, ER stress has been shown to activate autophagy in mammalian cells through signaling crosstalk between autophagy and canonical UPR pathways [201;202]. Animal studies have shown that ER stress or oxidative stress induces adaptive autophagy upregulation in the early phase, which helps restoring intracellular homeostasis by disposing a number of harmful molecules such as unfolded or misfolded proteins in ER lumen, cytosolic proteins damaged by ROS, or even dysfunctional ERs and mitochondria [203-205]. However, when intracellular stresses remain unresolved, prolonged autophagy upregulation progresses into autophagy defect [139;140]. Given that ER stress pathway is closely linked to proinflammatory pathways mediated by IKKβ/NF-κB [196-198] or JNK [199], it is logical to predict that autophagy changes are linked to these inflammatory pathways. In parallel, autophagy pathway can relate to proinflammatory signaling via oxidative stress pathway, as shown by a very recent report [206] that autophagic counteraction of intracellular oxidative stress can suppress cellular inflammation by inhibiting oxidative stress-induced NLRP3 inflammasome activation. Indeed, recent literature has shown that autophagy defect can induce NF-κB-mediated inflammation in association with the development of cancer or inflammatory diseases (e.g., Crohn's disease) [207-211].

The connection between autophagy defect and proinflammatory activation of NF-κB pathway can also be inferred in metabolic syndrome, since both autophagy defect [126-133;200] and NF-κB activation [20-33] are implicated in the development of overnutrition-related metabolic diseases. This notion was recently experimentally proved in the CNS [40]. Mice with hypothalamic knockdown of autophagy-related protein-7 (Atg7) developed hypothalamic autophagy defect and concomitant activation of hypothalamic IKKβ/NF-κB pathway. Moreover, hypothalamic autophagy defect can promote inflammatory activation to exacerbate the development of HFD-induced obesity and metabolic comorbidities [40]. The linear relationship from autophagy defect to NF-κB-mediated metabolic inflammation was demonstrated by the observation that hypothalamus-specific IKKβ ablation abolished the deleterious effects of hypothalamic autophagy defect on central metabolic regulations [40].

### Brain immune receptors and IKKβ/NF-κB signaling in metabolic syndrome

Both TLR pathway and cytokine receptor pathways are closely related to IKKβ/NF-κB signaling in the central pathogenesis of metabolic syndrome. Overnutrition, especially in the form of HFD feeding, was shown to activate TLR4 signaling and downstream IKKβ/NF-κB pathway [36;37], leading to metabolic derangements such as central leptin resistance, systemic glucose intolerance, and weight gain [37]. In the study by Kleinridders et al [37], the critical involvement of TLR4 signaling was demonstrated through brain-specific deletion of myeloid differentiation factor 88 (MyD88) – an essential signaling adaptor for TLR pathways to activate downstream proinflammatory signaling mediated by IKKβ/NF-κB or JNKs [212;213]. However, inhibition of central TLR4 signaling through brain-specific MyD88 deletion only abolished HFD-induced IKKβ/NF-κB activation but not JNK activation in mouse hypothalamus [37], suggesting that differential upstream signaling mechanisms exist for different proinflammatory kinase pathways in central metabolic inflammation. Interestingly, Gorina et al [214] reported a similar signaling interplay in astrocyte inflammation, i.e., TLR4 activation leads to MyD88-dependent NF-κB activation in early phase and MyD88-indepdnent MAPK/JNK pathway in late phase. Together these studies point to NF-κB as an immediate signaling effector for TLR4 activation in central inflammatory response. In addition to directly activating proinflammatory kinase pathways upon overnutrition, TLR4 activation has been shown to induce intracellular ER stress to indirectly cause metabolic inflammation in the hypothalamus [36;105]. Thus, central TLR4-NF-κB pathway may represent one of the early receptor-mediated events in overnutrition-induced central inflammation.

The close link between IKKβ/NF-κB and cytokine receptor signaling in metabolic inflammation is beyond doubt, given that many cytokines and their receptors are both upstream activating components and downstream transcriptional targets of NF-κB activation [176]. For example, central administration of TNF-α at low dose can mimic the effect of obesity-related inflammatory milieu to activate IKKβ/NF-κB proinflammatory pathways, furthering the development of overeating, energy expenditure decrease, and weight gain [34;159]. However, the physiological effects of IKKβ/NF-κB activation seem to be cell type-dependent, i.e., IKKβ/NF-κB activation in hypothalamic agouti-related protein (AGRP) neurons primarily leads to the development of energy imbalance and obesity [34]; while in hypothalamic POMC neurons, it primarily results in the development of hypertension and glucose intolerance [38;39]. Thus, cautions should be taken when inferring the biological effects of cytokine receptor pathways in central metabolic inflammation. IKKβ/NF-κB activation has also been linked to cytokine receptor-mediated inflammatory signaling in non-neuronal cells. For instance, central administration of interleukin-4 can induce microglial activation to promote the development of hypothalamic inflammation and resulting weight gain, yet these effects are abolished by central administration of IKKβ inhibitor [215]. Therefore, cytokine receptor signaling in glial cells may join neuronal inflammation, possibly via a paracrine mechanism, to cause the central dysregulation of metabolic physiology. The crosstalk between glial and neuronal cells in central metabolic inflammation still represents an under-investigated topic.

### Brain stress and therapeutic applications in obesity

The CNS, particularly the hypothalamus, is the central regulator of energy and body weight balance [47-62]. This regulation critically depends on neurons that are located at different hypothalamic metabolic sensing centers, which through regulated release of neuropeptides and neurotransmitters [216-222], control downstream neuroendocrine and neural systems to affect feeding and energy expenditure. The ability to properly detect whole-body energy and nutrient states, i.e., metabolic sensing, is crucial for these first-order hypothalamic neurons to manage energy balance. At the molecular level, metabolic sensing of neurons is mediated critically by canonical leptin signaling via JAK2/STAT3 pathway and insulin signaling via PI3K/Akt pathway [47;53;58]. However, under pathological conditions such as overnutrition-induced intracellular metabolic stresses, neuronal proinflammatory pathways are activated, which in turn impairs leptin and insulin signaling, leading to neuronal dysfunction and central body weight dysregulation [223-230]. For example, HFD feeding-induced activation of IKKβ/NF-κB proinflammatory pathway in the hypothalamus [34-37;40], whether its upstream signaling event being ER stress [34;36], autophagy defect [40], or TLR activation [36;37], ultimately leads to increased energy intake, decreased energy expenditure, and the development of obesity. Accordingly, inhibition of hypothalamic IKKβ/NF-κB signaling effectively protects against these metabolic disorders, as shown by various experimental animal models including pharmacologic inhibition of hypothalamic IKKβ [35], brain-specific deletion of IKKβ [34], IKKβ/NF-κB signaling effector SOCS3 [45;231] or TLR4 signaling adaptor MyD88 [37], or whole-body genetic deficiency of NF-κB subunit p50 [232] or TLR4 [36;154]. In fact, the therapeutic effect of anti-inflammation against obesity condition has been demonstrated in human subjects as well. In a retrospective case-control study, anti-inflammatory intervention with aspirin was shown to significantly promote weight loss in patients with T2D [233]. Also, the appetite-suppressing and anti-weight gain effects of rimonabant are associated with systemic decrease of inflammatory response [234].

### Brain stress and therapeutic applications in diabetes

The hypothalamus plays a central role in controlling glucose homeostasis through coordinating the regulatory networks formed between multiple organs including brain, liver, pancreas, adipocytes, and skeletal muscles [55;235-239]. Specifically, metabolic signals such as circulating leptin, insulin, gut hormone, and nutrients act on certain hypothalamic neurons to inform the brain of whole-body glucose homeostatic state. These neurons in turn generate appropriate metabolic orders through downstream neuroendocrine and neural systems to control peripheral glucose metabolism [240]. In particular, AGRP neurons and POMC neurons of the arcuate nucleus in mediobasal hypothalamus and steroidogenic factor 1 (SF1) neurons in ventromedial hypothalamus have been recently identified to employ leptin and insulin signaling to regulate peripheral glucose homeostasis [241-244]. Resembling the central dysregulation of energy balance, overnutrition-induced intracellular stresses and the ensuing cellular inflammation impair the normal metabolic signaling in these glucose-regulating neurons [245-248], leading to central dysregulation of glucose homeostasis [249-251]. On the contrary, stress counteraction has been shown as an effective therapeutic strategy against overnutrition-related glucose disorders in a battery of animal models. For example, ameliorating ER stress of obese mice through genetic overexpression of UPR components ATF6 [252], XBP1 [194], or ER chaperone proteins GRP78 [253], ORP150 [254], or pharmacologic administration of ER stress inhibitor tauroursodeoxycholic acid (TUDCA) [195], all improved glucose tolerance, insulin signaling, and related lipid disorders. More excitingly, this organelle-specific therapy has proven effective for human T2D [255-257]. For example, TUDCA can improve liver and muscle insulin sensitivity by approximately 30% in obese men and women [258]. Stavudine, an antioxidant molecule that reduces ROS production and enhances mitochondrial function, can also increase muscle insulin sensitivity in humans [259]. Additionally, anti-inflammation medications such as aspirin [260] and salsalate [261;262] have proven effective against T2D and related lipid disorders in clinical trials. Similarly, antagonizing systemic proinflammatory factors such as interleukin-1 [263] or TNF-α [264;265] also showed therapeutic effects against T2D.

### Brain stress and therapeutic applications in CVDs

Many clinical and epidemiologic studies have demonstrated the therapeutic/preventive effects of anti-cellular stress agents against CVDs. For example, dietary supplementation of antioxidant vitamin E can reduce the development of atherosclerosis through increased production of oxidation-resistant low-density lipoprotein [266]. Antioxidant compounds that can reduce cellular oxidative stress and/or enhance mitochondrial respiratory function, such as coenzyme Q10, α-lipoic acid, and α-L-carnitine, have been shown to protect against myocardial dysfunction [267] or improve systolic blood pressure in patients with coronary artery disease [268]. Moreover, an increasing number of new cardiovascular drugs are being developed which belong to the same antioxidant category [269]. More recently, suppressing ER stress has been proposed as a potential treatment strategy against myocardial infarction and heart failure [99]. Brain stress, being a primary pathogenic basis of metabolic syndrome, conceivably can underlie the development of metabolic syndrome related CVDs. However, because the concept of brain stress in metabolic syndrome is a relatively recent establishment, there have not been many mechanistic studies which directly demonstrate their causal relationship or the therapeutic potentials of inhibiting brain stress in CVDs. Nonetheless, two very recent animal studies have pointed to this possibility [38;39]. Overnutrition-related metabolic inflammation in the hypothalamus, specifically in POMC neurons of the arcuate nucleus, was found to underlie the development of obesity-related hypertension in mice [38]. Furthermore, POMC neuron-specific inhibition of this inflammatory pathway was shown to protect against the development of hypertension despite co-existing obesity or obesogenic condition [38]. In a following study [39] by the same group, brain ER stress was identified as the event upstream of hypothalamic NF-κB activation in the development of central inflammation-induced hypertension, and suppressing brain ER stress effectively prevented the development of overnutrition-induced blood pressure disorders.

## CONCLUDING REMARKS

Research in the past decade has established that metabolic syndrome can result from innate immune activation in response to overnutrition. While this type of inflammation exists broadly across different tissues, the CNS is in a primary and wide-impact position for the induction of metabolic syndrome by nutritional inflammation. An inflammatory state in brain regulatory centers such as the hypothalamus disrupts its metabolic sensing function, which in turn affects downstream neural and neuroendocrine regulation of a wide range of physiological processes such as energy balance, glucose metabolism, and cardiovascular homeostasis. Dysregulations of these processes often happen concurrently and manifest as a cluster of highly associated metabolic disorders such as obesity, insulin resistance, and hypertension. Research in the past few years has significantly differentiated the hypothalamic inflammatory pathways underlying these metabolic disorders, and overnutrition-induced intracellular stresses have been recognized as key activators of metabolic inflammation in the hypothalamus. This new knowledge not only provides a conceptual framework for further dissecting the pathogenesis of metabolic syndrome related diseases, but also indicates potential interventional strategies of counteracting neuroinflammation against metabolic diseases. Regardless of this exciting status quo, many important questions still remain to be addressed experimentally. It is fair to say that current understandings on the central inflammatory mechanisms of metabolic syndrome and related diseases are still in a primitive stage. However, in light of its great significance from both biomedical research and therapeutic application perspectives, we expect major research endeavors being drawn to this field and more advances being made in the near future. We also anticipate that eventually these findings will be translated into novel and effective treatments/preventions against miscellaneous overnutrition-induced metabolic diseases.
